# Addressing chronic pain with Focused Acceptance and Commitment Therapy in integrated primary care: findings from a mixed methods pilot randomized controlled trial

**DOI:** 10.1186/s12875-022-01690-2

**Published:** 2022-04-14

**Authors:** Kathryn E. Kanzler, Patricia J. Robinson, Donald D. McGeary, Jim Mintz, Lisa Smith Kilpela, Erin P. Finley, Cindy McGeary, Eliot J. Lopez, Dawn Velligan, Mariana Munante, Joel Tsevat, Brittany Houston, Charles W. Mathias, Jennifer Sharpe Potter, Jacqueline Pugh

**Affiliations:** 1 Center for Research to Advance Community Health (ReACH), The University of Texas Health Science Center at San Antonio, 7703 Floyd Curl Dr., Mail Code 7768, San Antonio, TX 78229 USA; 2 Department of Psychiatry & Behavioral Sciences, Joe R. and Teresa Lozano Long School of Medicine, The University of Texas Health Science Center at San Antonio, San Antonio, TX USA; 3 Department of Family & Community Medicine, Joe R. and Teresa Lozano Long School of Medicine, The University of Texas Health Science Center at San Antonio, San Antonio, TX USA; 4 Mountainview Consulting Group, Inc, Portland, OR USA; 5 Department of Rehabilitation Medicine, Joe R. and Teresa Lozano Long School of Medicine, The University of Texas Health Science Center at San Antonio, San Antonio, TX USA; 6 Barshop Institute for Longevity and Aging Studies, The University of Texas Health Science Center at San Antonio, San Antonio, TX USA; 7 Los Angeles Veterans Health Care System, Los Angeles, CA USA; 8 Department of Medicine, Joe R. and Teresa Lozano Long School of Medicine, University of Texas Health Science Center San Antonio, San Antonio, TX USA; 9 U.S. Army Medical Department (AMEDD) Quality and Safety Center, US Army Medical Command, San Antonio, TX USA; 10 Department of Population Health, Dell Medical School, The University of Texas at Austin, Austin, TX USA

**Keywords:** Chronic pain, Primary health care, Behavioral health consultation, Primary care behavioral health, General practice, Mixed methods, Acceptance and commitment therapy

## Abstract

**Background:**

Over 100 million Americans have chronic pain and most obtain their treatment in primary care clinics. However, evidence-based behavioral treatments targeting pain-related disability are not typically provided in these settings. Therefore, this study sought to: 1) evaluate implementation of a brief evidence-based treatment, Focused Acceptance and Commitment Therapy (FACT-CP), delivered by an integrated behavioral health consultant (BHC) in primary care; and 2) preliminarily explore primary (self-reported physical disability) and secondary treatment outcomes (chronic pain acceptance and engagement in valued activities).

**Methods:**

This mixed-methods pilot randomized controlled trial included twenty-six participants with non-cancer chronic pain being treated in primary care (54% women; 46% Hispanic/Latino). Active participants completed a 30-min individual FACT-CP visit followed by 3 weekly 60-min group visits and a booster visit 2 months later. An enhanced treatment as usual (ETAU) control group received 4 handouts about pain management based in cognitive-behavioral science. Follow-up research visits occurred during and after treatment, at 12 weeks (booster visit), and at 6 months. Semi-structured interviews were conducted to collect qualitative data after the last research visit. General linear mixed regression models with repeated measures explored primary and secondary outcomes.

**Results:**

The study design and FACT-CP intervention were feasible and acceptable. Quantitative analyses indicate at 6-month follow-up, self-reported physical disability significantly improved pre-post within the FACT-CP arm (*d* = 0.64); engagement in valued activities significantly improved within both the FACT-CP (*d* = 0.70) and ETAU arms (*d* = 0.51); and chronic pain acceptance was the only outcome significantly different between arms (*d* = 1.04), increased in the FACT-CP arm and decreased in the ETAU arm. Qualitative data analyses reflected that FACT-CP participants reported acquiring skills for learning to live with pain, consistent with increased chronic pain acceptance.

**Conclusion:**

Findings support that FACT-CP was acceptable for patients with chronic pain and feasible for delivery in a primary care setting by a BHC. Results provide preliminary evidence for improved physical functioning after FACT-CP treatment. A larger pragmatic trial is warranted, with a design based on data gathered in this pilot.

**Trial registration:**

clinicaltrials.gov, NCT04978961 (27/07/2021).

## Introduction

Most of the 100 million Americans with chronic pain receive treatment in primary care settings [1, 2]. Despite effective behavioral/nonpharmacologic interventions for chronic pain, patients continue to be offered primarily biomedical treatments, [3] rather than the full range of treatments that work [4].

Delivering behavioral treatments in primary care settings improves access to care for chronic pain. The Primary Care Behavioral Health model [5–7] increases availability of behavioral treatments by utilizing trained Behavioral Health Consultants (BHCs)—usually clinical psychologists or clinical social workers—who serve as members of the primary care team and deliver evidence-based nonpharmacologic treatments to patients with medical and/or psychological concerns. BHCs draw from a variety of evidence-based treatments to deliver brief, focused interventions to improve patient functioning [8–10].

One study to date examined BHC-delivered pain treatment using Brief Cognitive Behavioral Therapy for Chronic Pain (Brief CBT-CP) [11] and found that patients with musculoskeletal pain reported significantly less pain intensity, fewer functional limitations and improved self-efficacy [12]. While Brief CBT-CP is promising, more nonpharmacologic treatments are needed to increase options for pain treatment in primary care.

Another form of CBT is Acceptance and Commitment Therapy (“ACT”), [13] which has a strong scientific basis for improving chronic pain outcomes [14–17]. In contrast to Brief CBT-CP and traditional CBT approaches, ACT emphasizes patient values and focuses on improving overall quality of life, eschewing traditional targets of pain intensity and pain control [17, 18]. ACT uses a variety of experiential techniques to help patients improve their acceptance of chronic pain, a core component of psychological flexibility, involving willingness to engage in meaningful activities while in the presence of difficult experiences (i.e., unwanted physical and emotional aspects of chronic pain) [19].

Although substantial evidence for the general efficacy and effectiveness of ACT abounds, only one study has examined ACT for chronic pain provided in a primary care setting [20]; study findings were promising, but the duration of the group-based treatment protocol (16 sessions) is likely to make it infeasible in many settings. However, ACT in a brief format is known as “Focused” ACT (FACT), and it may be particularly suited for delivery by BHCs due to its succinct and flexible approach [21, 22]. However, no studies have examined FACT for chronic pain delivered by BHCs in a primary care setting.

Therefore, we developed a brief (5-visit) FACT for chronic pain (“FACT-CP”) -based group protocol and conducted a pilot randomized controlled trial (RCT) in a “real world” primary care setting. The objectives of this paper are to report on findings from our study and to further describe our methods and analyses. Details of the rationale, methods, and intervention are published elsewhere [23]. In preparation for a larger pragmatic trial in the future [24, 25], the aims of this pilot study were to:

1. Determine feasibility, acceptability and preliminary effectiveness of a FACT-CP treatment protocol for chronic pain delivered by a BHC in primary care, assessing patients pre- and post-treatment (at booster visit/12 weeks) and at 6-month follow-up.
2. Explore underlying secondary outcomes, including chronic pain acceptance and engagement in values-based activity, in FACT-CP participants.
3. Gather qualitative data to understand experiences of study participants and perceived benefits of the intervention in order to inform a larger trial and future implementation efforts.

## Methods

### Study design

This study was a mixed-methods sequential explanatory [26] pilot RCT with 6-month follow-up. The complete protocol, including description of the study design and methods was published prospectively [23] and registered retrospectively (27/07/2021) at ClinicalTrials.gov, #NCT04978961. All procedures were approved by the Institutional Review Board at The University of Texas Health Science Center at San Antonio (HSC20160512H) in accordance with federal codes for the conduct and protection of human subjects.

### Setting and participants

Our study site was a primary care clinic affiliated with The University of Texas Health Science Center at San Antonio using the Primary Care Behavioral Health model [7] of integrated care; the census in the clinic at the time was approximately 6500 unique patients. The study site had one clinical half-time BHC and one study-funded BHC (approximately 0.1 FTE) who conducted all of the clinical procedures detailed below. Patient inclusion criteria required (a) age 18 and older; (b) at least one non-cancer pain condition persisting for 12 weeks or more; (c) a current primary care clinician at the study clinic; and (d) ongoing treatment for a non-cancer chronic pain condition. Exclusion criteria were minimized for generalizability: (a) social anxiety or unwillingness to participate in a class setting; (b) presence of symptoms of psychosis and/or delirium; (c) a medical condition or life circumstance that would contraindicate or prevent participating (e.g. upcoming surgery); and (d) inability to comprehend the informed consent process or study instructions.

### Recruitment

Patients were recruited via: advertisements about the study posted in the clinic; referral by clinic personnel; and direct invitation based on pre-screening eligibility identified in the electronic health record. Recruitment and treatment were rolling, such that participants were enrolled as they completed pre-screening and consent procedures. Potential participants who indicated interest were pre-screened for eligibility by study staff then scheduled for their first visit where they completed informed consent procedures and baseline assessments. Participants were compensated for attending assessment visits. We also held a raffle for $10 gift card at each FACT-CP group visit to enhance retention. FACT-CP treatment was provided at no cost. This study started enrollment in October, 2017, and 6-month follow-up assessments concluded in December, 2018; exit interviews concluded in April, 2019.

### Randomization

**Fig. 1:**
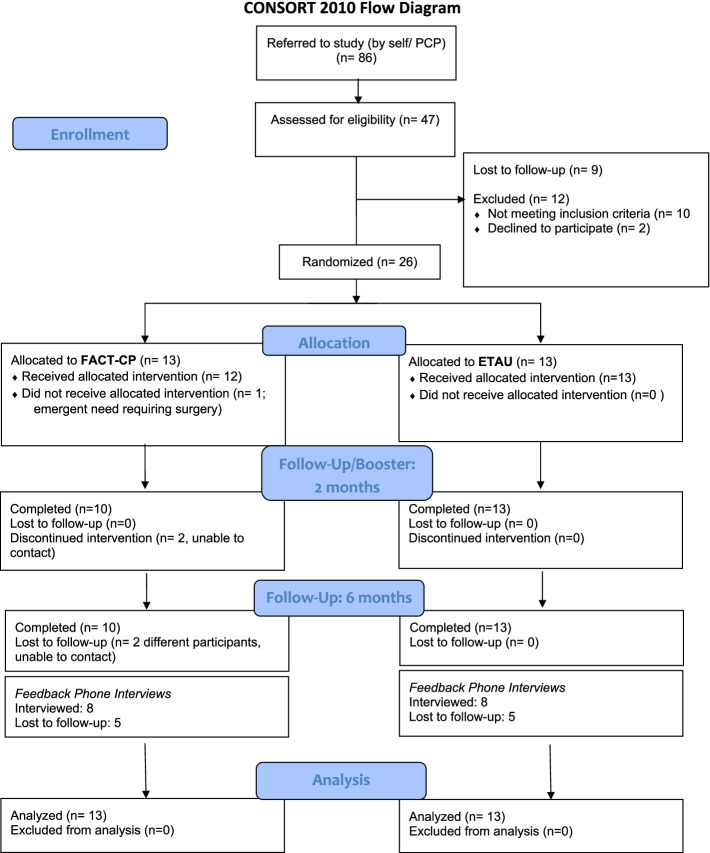
CONSORT Chart. FACT-CP: Focused Acceptance and Commitment Therapy; ETAU: Enhanced treatment as usual

Following prescreening, consent, and baseline assessment, participants were randomized to receive either FACT-CP or Enhanced Treatment as Usual (ETAU). Because pain severity is a factor that could affect outcomes and/or responsiveness to intervention [27], we stratified to avoid misattributing effects of the intervention (i.e., type 1 error), which is especially important in small trials [28]. At baseline, participants rated their level of pain severity using the *Numeric Rating Scale (NRS) for Pain*, a frequently-used single-item measure consisting of a horizontal line anchored with numeric labels (0 = no pain and 10 = worst possible pain). We developed 3 levels of stratification based on clinical consensus in our team and previously-recommended cut-points [29, 30]: 1–3 (mild), 4–7 (moderate), and 8–10 (severe). Using SAS software (Cary, NC) we created randomized block sizes ranging from 4 to 12. Varying sizes were used to balance groups while blinding study staff/PI to assignment, with NRS score category entered into a custom web-based application to facilitate and mask the randomization process (see Fig. 1 for our CONSORT diagram) [31].

### Data collection

**Fig. 2:**
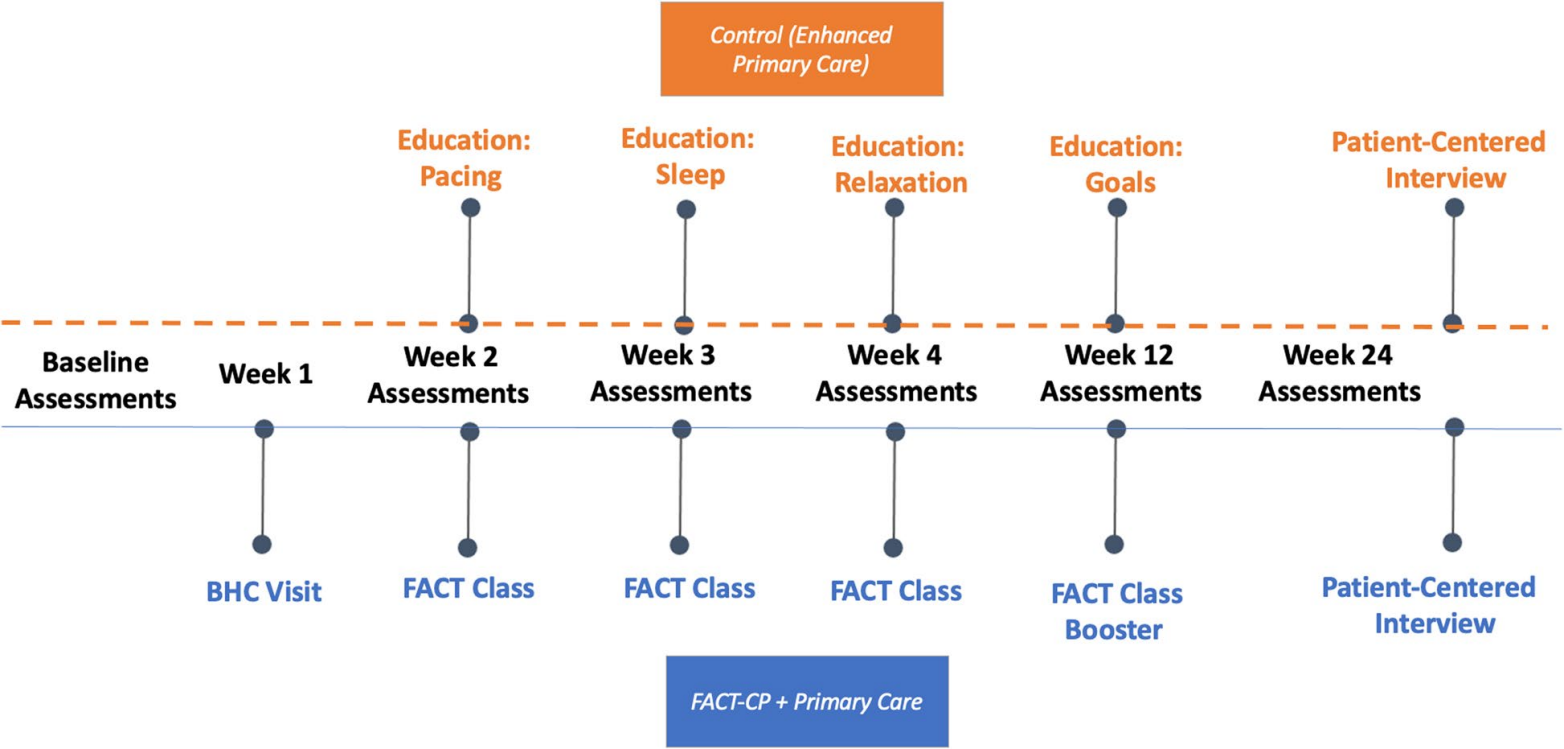
Treatment and research assessment visit timeline. FACT-CP: Focused Acceptance and Commitment Therapy; ETAU: Enhanced treatment as usual

Self-report measures described below were administered at 6 assessment visits: baseline, weeks 2, 3, 4, 12 (booster) and 24 (6-month follow-up; see Fig. 2). The study-appointed BHC was not involved in these visits and was blind to assessment results throughout. The study BHC did administer screening measures at each FACT-CP visit, including the NRS, in keeping with usual practice and standards of care in the clinic*.* Additional data were collected after the 6-month assessment visit by the PI (KEK), who contacted participants via phone and conducted semi-structured “exit interviews.”

### Interventions

#### Focused Acceptance and Commitment Therapy treatment

Approximately 1 week after baseline quantitative assessment, participants in the FACT-CP arm attended a 30-min individual visit with the BHC consisting of typical initial visit activities [7], including role clarification of the BHC; contextual interview for functional analysis of the pain problem; biopsychosocial case conceptualization; the brief FACT-CP intervention; between-visit exercise recommendations; and collaboratively-developed goals.

Over the next 3 weeks, participants attended weekly 1-h group visits focused on increasing acceptance of chronic pain towards greater psychological flexibility in responding to and coping with chronic pain, followed by a “booster” visit approximately 2 months later [23]. The BHC communicated with participants’ primary care clinicians throughout the course of FACT-CP care (individual BHC visit through booster visit) and kept clinical notes in their electronic health records, consistent with usual practice.

#### Enhanced treatment as usual

Participants assigned to ETAU attended assessment visits where they completed study measures and then received a 1-page double-sided handout from the study research assistant that was based on CBT principles for coping with stress and pain (relaxation, pacing, sleep, goal-setting). Participants in both arms continued to access usual primary and specialty care treatment throughout the study.

### Sample size

Recruitment was stopped at 13 per arm, in keeping with guidance on conducting pilot and feasibility studies [24, 25] (e.g., CONSORT [31]). We initially sought a larger sample size, however, at this stage, our team determined that a smaller sample was sufficient to pilot our goals examining feasibility and acceptability of the interventions, as well as study procedures, and to obtain preliminary data on the effectiveness of the FACT-CP intervention.

### Primary outcome measures (quantitative data)

#### Feasibility

Feasibility was evaluated using a priori established benchmarks: (a) < 25% participant attrition; (b) at least 80% of participants rating the FACT-CP program as satisfactory as measured by response of at least 5 on a 7-point Likert-scale (1 = Not Satisfied at All, 7 = Very Satisfied). The satisfaction question was asked in the context of an “exit interview”: all participants who completed the 6-month follow-up assessment were contacted by the PI to provide feedback on their experiences in the study. The semi-structured phone interview lasted approximately 10 min and the quantitative data portion included 5 Likert-scale questions to assess patient experiences with study participation, ease or difficulty of learning pain management skills, amount of information learned, and satisfaction with treatment.

Feasibility measures also included fidelity checks of the study BHC. Fidelity to FACT-CP was independently assessed by the study’s external consultant (PR). All treatment visits were audiotaped. Our consultant randomly selected and listened to 22% of these visits, assessing fidelity using a standardized checklist based on the FACT-CP treatment manual. Fidelity was evidenced by greater than 95% adherence to treatment.

#### Acceptability

Acceptability of the FACT-CP intervention from the participants’ perspective was measured via 3 Likert-scale questions gathered during the semi-structured interview: perceived benefit, ease of learning about pain management, and whether the participant would recommend the FACT-CP treatment to a friend or family member.

#### Effectiveness

The primary outcome of effectiveness was self-reported physical disability, assessed using the modified and psychometrically sound *Oswestry Disability Index* (ODI) [32]. The ODI is a 10-item self-report measure using 6-point Likert scales, originally developed as a measure of back pain. We used an established modified version that asked about “pain” rather than “back pain” [33, 34]. Scores are summed to create a total score (maximum 50) that is then divided by the highest possible score based on items completed, then doubled to provide a percentage of disability. Reliability in our study was high (Cronbach’s alpha, α = .85).

### Secondary outcome measures

Pain acceptance was examined using the *Chronic Pain Acceptance Questionnaire-Revised (CPAQ)* [35]*.* The 20-item CPAQ assesses the degree to which chronic pain and related experiences influence behaviors and the degree of effort put in to controlling pain. Items are responded to on a 0 to 6 Likert scale. Higher scores indicate greater acceptance; scores range from 0 to 120. We measured engagement in values-based activity with the *Chronic Pain Values Inventory* (CPVI) [36, 18], an inventory that identifies which values are important to a patient with chronic pain, and assesses the degree of success they are having in following their values. The valued domains are family, intimate relations, friends, work, health, and growth or learning. The 12-item CPVI uses 6-point Likert scale questions to measure the discrepancy between ratings of importance of valued life areas and success in engaging in those life areas; lower scores reflect greater alignment (i.e., less discrepancy) between values and actions in one’s life. The stand-alone success scale includes ratings on the engagement items only. We chose to employ the discrepancy scale rather than the success scale because we wanted to measure success in engagement in valued activities in the context of their perceived importance. CPVI scores range from 0 to 6. Reliability was high for both the CPAQ (α = .85) and the CPVI (α = .82).

### Measures of participants’ experiences (qualitative data)

Qualitative data were also gathered during the exit interviews, which included open-ended questions assessing the following domains: what participants liked most and least about their participation, and any changes in pain management or quality of life due to participation (e.g., “In what ways has your participation in our study changed the way you think about or manage pain?”). Participants were given time to discuss anything else they wanted to share with the PI. The PI took near-verbatim contemporaneous notes during the interviews.

### Analyses

#### Aim 1 analyses

We examined acceptability and feasibility of FACT-CP and study procedures by calculating percentages and frequencies. Physical disability (primary outcome) was examined using a general linear mixed (within and between groups) regression model with repeated measures, controlling for baseline pain severity, with the primary focus on comparing pre-post change in the 2 study arms. Fixed effects in these statistical models were treatment arm, time, and the treatment-by-time interaction. Although the analysis does produce conventional ANOVA-type tests, those are non-specific. Instead, the hypothesis tests were done using planned, a priori contrasts that compare the regression-based least-square means to estimate change in a group using all subjects (intent-to-treat analysis), including those with missing data. Baseline pain severity was included as a covariate because it was used to stratify randomization [37].

#### Aim 2 analyses

We examined secondary outcomes, acceptance of chronic pain (CPAQ) and engagement in values-based activity (CPVI) between and within groups using general linear mixed regression models with repeated measures, again with the primary focus on comparing pre-post change in the 2 study arms. Baseline pain severity was again included as a covariate.

Missing data analyses.

Across the 6 weeks of assessments, between 0 and 15.4% of data were missing from each measure. Missing data were handled using maximum likelihood estimation. This yields valid parameters given the usual assumption that data are missing at random. Additionally, Little’s MCAR test was non-significant for all variables, [ODI: *X*^2^ (15, *N* = 26) = 8.34, *p* = .909; CPAQ: *X*^2^ (11, *N* = 26) = 8.13, *p* = .702; CPVI: *X*^2^ (15, *N* = 26) = 14.08, *p* = .520]. All data were analyzed using SPSS 26.0 [38] and/or SAS v9.4 (Cary, NC).

#### Aim 3 qualitative analyses

We analyzed qualitative interview data using rapid qualitative analysis [39, 40]. This approach, compared with in-depth analyses, is particularly useful in studies with resource constraints (i.e., a pilot study) [41] and in research conducted in clinical settings, to aid in timely dissemination of patient feedback [39–41]. Rapid qualitative analyses has been compared directly with more traditional thematic analysis and found to produce closely aligned results, and is thus considered to have comparable rigor [40]. Participant responses were organized by question (domains for both groups: Best/Most Likeable Features; Worst/Most Disliked Features; Changes in Perception of Pain; Changes in Quality of Life; and Other Feedback). Analyses were structured to identify similarities and differences within and between groups. Interview notes were consolidated into a matrix, with rows for each domain and columns for individual respondents. Themes emerging within each domain and exemplar quotes were then identified by 2 co-authors who met in person to reach consensus (KEK and EPF); 3 other independent raters (BH, LSK, CM) reviewed the table of consolidated themes and quotes via email and/or in-person review and provided iterative feedback until consensus was achieved [40].

## Results

### Sample characteristics

Participants’ average age was 52 years, more than half were women (53%), and most identified their race as white (85.8%), followed by “other” (11.5%) and Asian (3.8%); Hispanic ethnicity was reported by 46.2% of the participants (Table 1). On average, participants had experienced chronic pain for 11.9 years, most commonly musculoskeletal pain in multiple sites (50%) or throughout the body (46%). There were no meaningful statistical differences between groups at baseline on demographic variables; physical disability level; or pain severity, duration or acceptance; but the ETAU group reported significantly greater discrepancies between ratings of importance of valued life areas and success in engaging in activities (CPVI).

**Table 1:** Demographics

	FACT *N* = 13	TAU *N* = 13	Total *N* = 26
Gender			
Female	61.5% (8)	46.2% (6)	53.8% (14)
Mean age (range)	54 (26–79)	50 (32–66)	52 (26–79)
Ethnicity			
Hispanic/Latino	46.2% (6)	46.2% (6)	46.2% (12)
Non-Hispanic/Latino	53.8% (7)	53.8% (7)	53.8% (14)
Race			
White	76.9% (10)	92.3% (12)	84.6% (22)
Other	15.4% (2)	7.7% (1)	11.5% (3)
Asian	7.7% (1)	0.0% (0)	3.8% (1)
Relationship status			
Married/living with partner	84.6% (11)	53.8% (7)	69.2% (18)
Single/divorced/widowed	7.7% (1)	30.8% (4)	19.2% (5)
In a relationship	7.7% (1)	15.4% (2)	11.5% (3)
Education			
GED/High School diploma	7.7% (1)	23.1% (3)	15.4% (4)
Some college/ Associate’s degree	30.8% (4)	23.1% (3)	27.0% (7)
4-year college degree	38.4% (5)	30.8% (4)	34.6% (9)
Master’s degree	23.1% (3)	23.1% (3)	23.0% (6)
Annual household income			
< $10,000	0.0% (0)	7.7% (1)	3.8% (1)
$10,000-20,000	15.4% (2)	7.7% (1)	11.5% (3)
$20,000–$50,000	30.8% (4)	53.8% (7)	42.3% (11)
$45,000-100,000	30.8% (4)	15.4% (2)	23.0% (6)
> $100,000	23.1% (3)	15.4% (2)	19.2% (5)
Pain Type			
Fibromyalgia	15.4% (2)	7.7% (1)	11.5% (3)
Musculoskeletal	46.1% (6)	53.8% (7)	50.0% (13)
Multi-type	23.1% (3)	30.8% (4)	27.0% (7)
Other	15.4% (2)	7.7% (1)	11.5% (3)
Pain Site			
Neck/Head	15.4% (2)	0.0% (0)	7.7% (2)
Upper Body	30.8% (4)	7.7% (1)	19.2% (5)
Lower Body	7.7% (1)	23.1% (3)	15.4% (4)
Back/Lower Back	7.7% (1)	15.4% (2)	11.5% (3)
Multi-Site/Whole Body	38.4% (5)	53.8% (7)	46.4% (12)
	Mean (SD)	Mean (SD)	*p*
Pain duration (years)	9.72 (7.38)	14.03 (13.07)	0.310
Pain severity, past 2 wks (NRS)	6.54 (1.90)	7.08 (1.55)	0.436
Physical disability (ODI)	38.02 (16.76)	43.57 (13.62)	0.363
Pain acceptance (CPAQ)	61.38 (15.08)	60.38 (18.53)	0.881
Values/activities discrepancy (CPVI)	1.73 (0.71)	2.34 (0.77)	0.045

*FACT* Focused Acceptance and Commitment Therapy, *ETAU* Enhanced treatment as usual, *ODI* Oswestry Disability Index, *CPAQ* Chronic Pain Acceptance Questionnaire, *CPVI* Chronic Pain Values Inventory (Discrepancy scale), *NRS* Numeric Rating Scale for Pain. No statistically significant differences were found between groups on any variables except CPVI

### Primary analyses

#### Feasibility

A priori benchmarks for feasibility were met for measures of retention, satisfaction, and fidelity. Retention in the study was demonstrated by absence of any treatment drop-outs during the initial FACT-CP intervention and 77% retention through the booster visit (see Fig. 1). 75% of patients reported satisfaction (rated at least 5 on the 7-point scale) with the FACT-CP intervention.

#### Acceptability

There were no statistically significant differences between groups in the 3 indicators of acceptability: perceived benefit, ease of learning, and recommendation of treatment (Table 2).

**Table 2:** Feasibility & Acceptability Ratings

	FACT-CP (mean; range; SD)	ETAU (mean; range; SD)
Satisfaction with treatment	75% (5; 1–7; 1.85)	88% (5.63; 4–7; 1.19)
Perceived benefit of treatment	88% (5.5; 2–7; 1.6)	75% (5; 1–7; 1.93)
Ease of learning pain management	88% (5.5, 1–7; 2)	88% (5.9; 3–7; 1.64)
Would recommend to others	100% (6.63; 6–7; 0.52)	88% (6.13; 2–7; 1.81)

Anchors were 1–7 (higher scores indicate a more positive response); percentage reflects those who rated items ≥5; no statistically significant differences were found between groups

#### Effectiveness of FACT-CP intervention

**Fig. 3:**
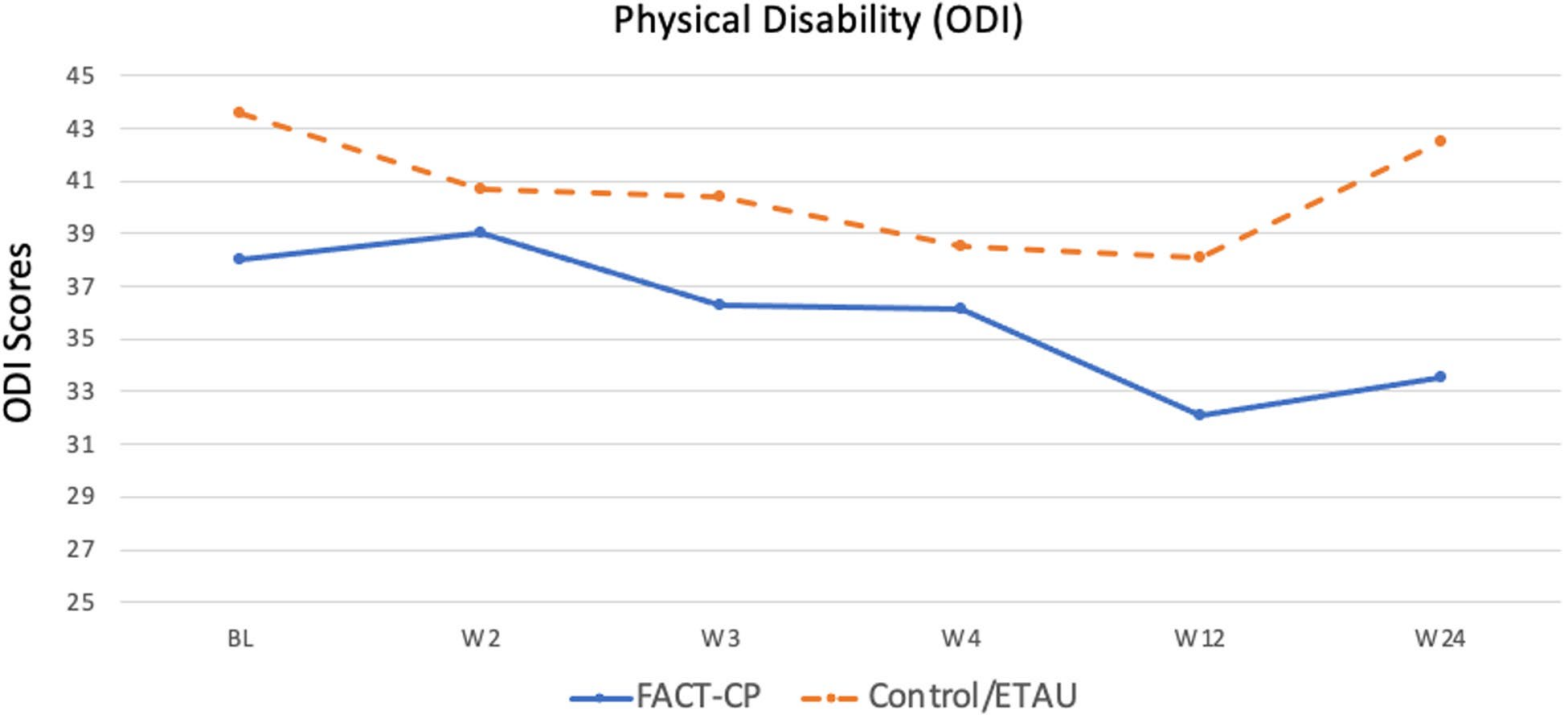
Changes in Physical Disability Over Time (Primary Outcome); ODI: Oswestry Disability Index; lower scores indicate better functioning

Physical disability significantly improved in the FACT-CP arm from baseline to booster visit at 12 weeks (*p* = 0.002) with a large effect size, *d* = 0.89, and at 6 months (*p* = 0.023) with a medium effect size, *d* = 0.64. The ETAU group also showed significant improvement from baseline to booster at 12 weeks (*p* = 0.003) with a large effect size, *d* = 0.84, but the improvement was no longer significant at 6-month follow-up (*p* = 0.546), *d* = 0.17. Differences between groups were not statistically significant after treatment at 12 weeks (*p* = 0.675), *d* = 0.16, or at 6-month follow-up (*p* = 0.196), *d* = 0.51 (see Fig. 3 and Table 3).

**Table 3:** Primary and secondary outcome measures

Variables & Arms	Baseline *M (SD)*	Post-treatment (Booster, 12 wks) *M (SD)*	*p*	*d* (95% CL)	6-month Follow-up *M (SD)*	*p*	*d* (95% CL)
**Acceptance (CPAQ)**							
*FACT-CP Arm (n = 13)*	61.39 (15.1)	61.7 (18)	0.512	0.18 (− 0.37, 0.73)	70.6 (17.52)	0.049	0.55 (0.00, 1.10)
*ETAU Arm (n = 13)*	60.39 (18.53)	56.23 (21.37)	0.147	−0.40 (− 0.95, 0.15)	55.39 (19.7)	0.082	− 0.49 (− 1.04, 0.06)
*Between-group differences*	–	–	0.145	0.58 (− 0.20, 1.35)	–	0.009	1.04 (0.26, 1.82)
**Disability (ODI)**							
*FACT-CP Arm (n = 13)*	38.02 (16.76)	28.89 (18.25)	0.002	−0.89 (−1.44, − 0.34)	31.53 (17.14)	0.023	−0.64 (− 1.19, − 0.09)
*ETAU Arm (n = 13)*	43.57 (13.62)	38.12 (17.8)	0.003	− 0.84 (− 1.39, − 0.29)	42.47.48 (20.60)	0.55	−0.17 (− 0.72, 0.38)
*Between-group differences*	–	–	0.675	− 0.17 (− 0.94, 0.61)	–	0.196	−0.51 (− 1.29, 0.27)
**Values/Action Discrepancy (CPVI)**							
*FACT-CP Arm (n = 13)*	1.73 (0.71)	0.87 (0.87)	0.0003	− 1.04 (− 1.59, − 0.49)	1.15 (0.85)	0.013	− 0.70 (− 1.25, − 0.15)
*ETAU Arm (n = 13)*	2.34 (0.77)	1.52 (0.81)	0.0009	− 0.94 (− 1.49, − 0.39)	1.90 (0.90)	0.068	−0.51 (− 1.06, 0.04)
*Between-group differences*	–	–	0.634	− 0.19 (− 0.96, 0.59)	–	0.545	−0.24 (− 1.02, 0.54)

*FACT-CP* Focused Acceptance and Commitment Therapy, *ETAU* Enhanced treatment as usual, *ODI* Oswestry Disability Index, *CPAQ* Chronic Pain Acceptance Questionnaire, *CPVI* Chronic Pain Values Inventory (Discrepancy scale)

### Exploratory analyses

#### Secondary outcomes

##### Chronic pain acceptance

**Fig. 4:**
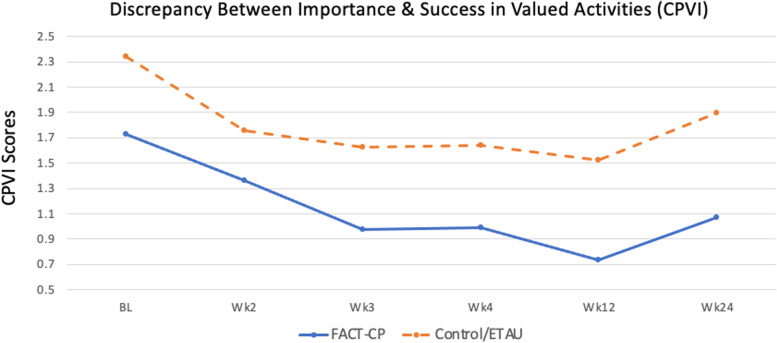
Chronic Pain Acceptance Over Time (Secondary Outcome); CPAQ: Chronic Pain Acceptance Questionnaire; higher scores indicate greater acceptance

Findings indicated that chronic pain acceptance (CPAQ) increased from baseline to 12 weeks (booster) for the FACT-CP group, and decreased for the control group, but these changes were not statistically significant (*p* = 0.51 and *p* = 0.147); there was a medium effect size for this difference, *d* = 0.58. However, by follow-up at 6 months, the FACT-CP arm had a significant increase in chronic pain acceptance from baseline (*p* = 0.049) with medium effect size, *d* = 0.55; and the ETAU arm experienced a significant decrease in chronic pain acceptance (*p* = 0.082) with a medium effect size *d* = − 0.49. This difference between groups was significant (*p* = 0.009) with a large effect size, *d* = 1.04 (see Fig. 4 and Table 3).

##### Valued activities

**Fig. 5:**
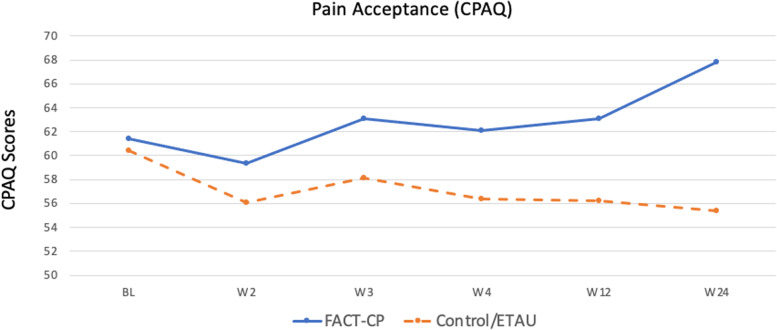
Changes in Valued Activities Over Time (Secondary Outcome); CPVI: Chronic Pain Values Inventory; Lower scores indicate less discrepancy between importance of values and success engaging valued activities

The discrepancy between importance and success in engaging in valued activities significantly decreased in the FACT-CP arm from pre- to post-treatment at 12 weeks (*p* = 0.0003) with a large effect size, *d* = 1.05, and at 6-month follow-up (*p* = 0.013) with a moderate-large effect size, *d* = 0.76. At 12-weeks post-treatment, the ETAU group also evidenced significantly decreased discrepancy scores (*p* = 0.0009) with a large effect size, *d* = 0.94; and at 6-month follow-up (*p* = 0.044), with a medium effect size, *d* = 0.51. The difference between groups in valued activities was not significant at post-treatment (*p* = 0.63), d = 0.19, or follow-up (*p* = 0.54), d = 0.24 (see Fig. 5 and Table 3).

### Qualitative analysis of participant experiences

The FACT-CP participants appreciated acquiring skills for learning to live with pain (see Table 4). They also appreciated practicing mindfulness and meditation, and receiving treatment in a small group setting. Some of the ETAU participants reported that their handouts provided helpful reminders about how to manage chronic pain. Features of the treatment that were most disliked by the FACT-CP group included the perceived small “dose” of the program; participants were interested in additional monthly classes to facilitate deeper learning and connection with others. Some ETAU participants reported that the handouts were ineffective in helping to manage pain.

**Table 4:** Qualitative Data

***Domains***	***Themes & Exemplar Quotes***	
	*FACT Arm Participants*	*ETAU Arm Participants*
**Best/Most Likeable Features**	**Learned to live with pain**	**Handouts as reminders of good pain management**
	*The [ACT] matrix – quite often, they [exercises & classes] were educational and revealing – about how I was thinking about handling the pain*	*Just having the info and written instructions and could study it and practice what was in there and there were some good ideas in there*
	**Mindfulness and meditation**	**Neutral - information was not new or helpful**
	*Trying to focus on other things other than the pain – breathe, meditate, all of it*	*Been kind of dealing with [pain] for a long time so a lot of these tips/techniques I already learned about – wasn’t really new*
	**Small Groups**	**Great Research Assistant**
	*I like that it was just 2 people in the class—more intimate/ personalized/ customized*	*[RA] was personable, remembered things – very flexible in scheduling*
**Worst/Most Disliked Features**	**Wanted greater dose**	**Ineffective intervention**
	*I wish I could have done more sessions. Need more practicing, more sessions*	*It was frustrating to just get the paper; just do breathing*
	**Paperwork and questionnaires**	**Paperwork and questionnaires**
	*Filling out the forms every time*	*I don’t know...filling out all the paperwork. Wasn’t that big a deal … just the same questions over and over*
	**Nothing**	**Nothing**
	*There wasn’t really anything I disliked … it was good, the mental learning as well as doing*	*[There was] nothing that I disliked. Thought it was good.*
**Changes in Thinking About or Managing Pain**	**Changed relationship with pain/how handle pain**	**Reinforced good self-management**
	*I know that pain isn’t always going to stop... you can learn to live with it using techniques...[the] mind is a powerful thing, so if you can help your mind to believe it, you can do*	*Improved a little bit. Made me give more thought to what I do and how I live. Doing prevention rather than treatment*
	**Hope**	**Not much changed**
	*There is a next chapter … this won’t go on forever … there are things I can do besides quit or give in*	*It was something that I was already using, I’m more of the type that would rather read up on it than sit at home and take meds, [so it] didn’t really affect me one way or another because I was doing it already*
**Impact on Quality of Life**	**Changed perspective**	**Motivation/accountability**
	*I was able to stop dwelling on pain and stop being sorry for myself, was able to look at things clearer*	*Making a choice every day [to follow goal] – I used to lay in bed a whole weekend. That subsided during the study. I had to think to myself, “do you want to be a 7 or a 1?!” on the questionnaires*
	**Acquisition of tools to help live with pain**	**Being more active/using skills**
	*[Mindfulness] helped me get rid of the thought process I was in*	*[I got] a little more active but at my own pace … I focused on a lot of relaxation and sleep. Normally I don’t focus on sleep as much as I need, don’t rest, get busy with projects and won’t let my body rest*
		**No changes**
		*It didn’t really change*
**Additional Feedback**	**Content of intervention**	**Benefits of research**
	*To me it was a good program and think it will help others as well*	*Happy to advance research. Even though I didn’t get what the other group got. [It was] still valuable*
	**Thoughts on medication**	**Need for more clinical services**
	*Meds aren’t everything, giving people strategies [is important], and different people need different things*	*People like me, we get lost*
	**Process**	
	*I was looking for excuses not to go places or do things. The intense sessions weekly – that was very helpful*	

When asked about changes in thinking about or managing pain, FACT-CP participants described a changed relationship with their pain and acquisition of different strategies for handling pain. Participants described a more hopeful outlook after treatment, with one participant noting “there is a next chapter.” The reference to a chapter is directly related to a metaphor and exercise used in the FACT-CP class. FACT-CP participants described positive changes in their quality of life as a result of participating in this study, including changed perspectives on their abilities, life and pain; one participant noted, *“*I was able to look at things clearer*.”*

FACT-CP participants also mentioned developing more coping skills or tools to live with pain, such as being able to observe thoughts and not get stuck in them (i.e., through cognitive defusion exercises and mindfulness practices). Interestingly, some ETAU participants also experienced a positive impact on their quality of life, reported as a result of both the intervention handouts and the regular assessments. Some felt motivated or more accountable to engage in healthy pain management strategies because they had to report on many aspects of functioning via our assessment battery, with one participant reporting she would motivate herself by asking, “Do you want to be a 7 or a 1?!” Others said their quality of life improved to some degree because of increased awareness about how to care for themselves (e.g., handouts on sleep, relaxation).

## Discussion

This mixed-methods RCT pilot study examined brief BHC-delivered FACT-CP for chronic pain in an integrated primary care setting. We found that our study design, as well as mode of delivery of FACT-CP, were both feasible and acceptable. Furthermore, this pilot study provides preliminary evidence for improved self-reported physical disability. Regarding secondary outcomes, values-based activities improved for participants in both arms but remained significantly better only in the FACT-CP arm at 6-month follow-up. Furthermore, acceptance of chronic pain significantly increased in the FACT-CP arm, but decreased in the control group, resulting in a statistically significant difference between arms by 6-month follow up despite the small sample size, with a correspondingly large effect size.

FACT-CP participants reported finding the FACT-CP classes to be beneficial, easy to learn, and would recommend FACT-CP treatment to a family or friend. All but 1 FACT-CP participant gave ratings of at least 5 on a 7-point scale on items about perceived benefit and ease of learning. The FACT-CP intervention also met criteria for feasibility, based on success in retention, fidelity, and satisfaction.

Another notable finding is that 4.5 h of FACT-CP delivered by a BHC in primary care was promising in reducing self-reported disability. This finding is consistent with other studies of ACT interventions for people with chronic pain [14, 42]. Our secondary outcomes, especially chronic pain acceptance, were found to be influenced by FACT-CP treatment. Evidence abounds that acceptance is a powerful mechanism in improving functioning and emotional health in people with chronic pain [43]; future research on FACT-CP should examine this further in studies that are more adequately powered to examine that hypothesis. FACT-CP participants reported appreciating skills for learning to live with pain, consistent with increased chronic pain acceptance.

It is encouraging that a brief intervention produced a large between-group effect size in chronic pain acceptance, comparable to medium-large effect sizes identified in much longer ACT interventions [43]. Our effect size for acceptance at 6-month follow-up was also much larger than the reported effect in the 16-h ACT intervention in primary care at 3-month follow-up.

Interestingly, the Brief CBT-PC demonstration project (the only study of BHC-delivered treatment to date) reported medium effect size (*d* = 0.65) at the third appointment in pain intensity and functional limitations, but improvements diminished over subsequent visits [12]. In contrast, FACT-CP produced a large effect size by the fourth visit *(d* = 0.89) with reduced, but sustained improvements at 6 months (*d* = 0.69). Both of these modalities for chronic pain in Primary Care Behavioral Health settings require additional study, but it is favorable that 2 low-intensity interventions may be effective in improving functioning for patients with chronic pain.

The primary limitation of our study is the small sample size. Therefore, findings about effect of the FACT-CP intervention on acceptance need to be interpreted cautiously. While it is encouraging that an underpowered pilot study detected a large effect size, that result needs to be replicated in a larger sample. However, as Moore and colleagues [25] recommend, our study exceeded recommendations for *N* = 12/arm as the optimal sample size in a pilot RCT, and meets sample size recommendations to assess feasibility and acceptability of the intervention [24, 25]. Additionally, findings may not be generalizable due to the sample size, as well as specific geographic location.

Another study limitation is that the BHC interventionist was the PI (KEK), which could bias results, as she has expertise and 15+ years of experience delivering acceptance-based treatments. However, the PI is not likely to be a source of bias, as she was blind to research outcome assessments through the study. Additionally, study staff, not the PI, delivered the ETAU handouts to control participants. Due to the PI’s expertise, it is possible our findings would not generalize if less-experienced BHCs were delivering the intervention. As such, we developed the FACT-CP manual so that future BHCs could adopt the protocol without extensive training or experience; in our planned upcoming trial, we will examine clinical experience as potential factor affecting implementation, including adoption and effectiveness. Having the PI deliver the intervention was also beneficial in facilitating refinement of the treatment protocol and study design in preparation for a larger trial. Future dissemination and implementation efforts will focus on facilitating adoption of FACT-CP by a wide range of BHCs in diverse primary care clinics.

An additional limitation involves qualitative data collection, as the interviews were not recorded and transcribed. However, the interviewer (PI) took contemporaneous notes with an effort to capture statements verbatim, as in a real-time transcription. Although this means our data may not be as refined, employing such an approach is common in pragmatic implementation studies [44], and in this pilot study, the procedure reduced costs and resources required to record and professionally transcribe these interviews [41].

A final limitation to consider is that all measures in this study were self-reported, and are thus subjective. Yet, patient perception of their own disability status, as gathered in this study with the ODI, is highly correlated with objective ratings of pain behavior and physical functioning [45]. Nonetheless, incorporating objective measures of outcomes, including behavioral measures (e.g., daily diary ratings, app use, family-observed data on engagement in valued activity), physical functioning measures (e.g., 6-min walk test, sit-to-stand tests), or other objective measures (e.g., fitness tracker data, prescribed opioid use reduction, healthcare utilization related to pain exacerbations) in future studies would provide additional information regarding the impact of the FACT-CP protocol.

We sought to balance scientific rigor with demands of a “real-world” primary care setting by including both pragmatic and explanatory trial elements [46], publishing our protocol a priori [23] and setting up opportunities for a future, larger trial. Although we did not formally gather primary care clinician feedback, there were multiple avenues for study personnel, clinic leadership, clinicians, and staff to provide feedback before, during, and after the study. Still, future research should prioritize stakeholder perspectives throughout the study.

Testing on a larger scale, across multiple sites, will allow for fully powered evaluation of the effectiveness of this brief FACT-CP intervention. Future research should also study this intervention in underserved, more diverse primary care populations. Feedback from ETAU participants highlights the need to consider alternatives to traditional RCTs when conducting clinical research, such as using a waitlist-control design so that all participants can experience the active treatment. It may also be useful to study adaptation of this group intervention to individual BHC visits, which may be more feasible in some clinical settings and was requested by some participants.

## Conclusions

Our mixed-methods pilot RCT demonstrates that FACT-CP delivered by a BHC in primary care is feasible and acceptable and may improve physical disability. Acceptance of chronic pain emerged as a strong treatment outcome and should be examined as a mechanism of change in adequately powered future research. A well-powered pragmatic trial is warranted, with modifications based on data gathered in this pilot study.

## Data Availability

The datasets used and/or analyzed during the current study are available from the corresponding author on reasonable request.

## Abbreviations

ACT: Acceptance and Commitment Therapy
CBT: Cognitive Behavioral Therapy
BCBT-CP: Brief Cognitive Behavioral Therapy for Chronic Pain
BHC: Behavioral Health Consultant
CPAQ: Chronic Pain Acceptance Questionnaire-Revised
CPVI: Chronic Pain Values Inventory
ETAU: Enhanced Treatment as Usual
ODI: Oswestry Disability Index
NRS: Numeric Rating Scale for Pain
RCT: Randomized controlled trial
